# Prevalence of Pathogenic Germline *DICER1* Variants in Young Individuals Thyroidectomised Due to Goitre – A National Danish Cohort

**DOI:** 10.3389/fendo.2021.727970

**Published:** 2021-09-06

**Authors:** Mays Altaraihi, Thomas van Overeem Hansen, Eric Santoni-Rugiu, Maria Rossing, Åse Krogh Rasmussen, Anne-Marie Gerdes, Karin Wadt

**Affiliations:** ^1^Department of Clinical Genetics, Copenhagen University Hospital, Rigshospitalet, Copenhagen, Denmark; ^2^Department of Pathology, Copenhagen University Hospital, Rigshospitalet, Copenhagen, Denmark; ^3^Center for Genomic Medicine, Copenhagen University Hospital, Rigshospitalet, Copenhagen, Denmark; ^4^Department of Endocrinology and Metabolism, Copenhagen University Hospital, Rigshospitalet, Copenhagen, Denmark

**Keywords:** goitre, thyroidectomy, hereditary cancer, DICER1 syndrome, DICER1 mutation, goiter

## Abstract

**Introduction:**

DICER1 syndrome encompasses a variety of benign and malignant manifestations including multinodular goitre, which is the most common manifestation among individuals carrying pathogenic *DICER1* variants. This is the first study estimating the prevalence of pathogenic *DICER1* variants in young individuals with multinodular goitre.

**Methods:**

Danish individuals diagnosed with nodular goitre based on thyroidectomy samples in 2001-2016 with the age limit at time of operation being ≤ 25 years were offered germline *DICER1* gene testing.

**Results:**

Six of 46 individuals, 13% (CI [3.3;22.7], p <0.05), diagnosed with nodular goitre on the basis of thyroidectomy samples under the age of 25 years had pathogenic germline variants in *DICER1*. They were found in different pathoanatomical nodular goitre cohorts i.e. nodular goitre (n=2), colloid nodular goitre (n=3) and hyperplastic nodular goitre (n=1).

**Conclusions:**

We recommend referral of patients thyroidectomised due to goitre aged <21 years and patients thyroidectomised due to goitre aged <25 years with a family history of goitre to genetic counselling. Patients of all ages thyroidectomised due to goitre, who are affected by another DICER1 manifestation should be referred to genetic counselling.

## Introduction

DICER1 syndrome is a rare autosomal dominant disorder predisposing individuals to development of both benign and malignant neoplasms. The *DICER1* gene encodes a ribonuclease involved in processing pre-microRNA (miRNA) to mature miRNA. Alterations in *DICER1* lead to dysregulation of miRNA production, which is associated with different tumour types (1).

The hallmark tumours of the DICER1 syndrome are pleuropulmonary blastoma and Sertoli-Leydig cell tumour, which both are rare tumours. Most known families with pathogenic *DICER1* variants have been identified through clinical findings of the hallmark tumours, which can cause ascertainment bias associated with the DICER1 syndrome.

Some of the other DICER1-related tumours include cystic nephroma, anaplastic renal sarcoma, Wilms tumour, differentiated thyroid carcinoma, gynandroblastoma, ciliary body medulloepithelioma, embryonal rhabdomyosarcoma and primary brain tumours such as pineoblastoma and pituitary blastoma.

However, the most common manifestation among pathogenic *DICER1* variant carriers is multinodular goitre (MNG) diagnosed in young individuals. The cumulative incidence of thyroidectomy due to MNG is estimated to be 44% in women and 14% in men with *DICER1* pathogenic variants by age 30 years in an American cohort (2). To date, no studies have investigated the prevalence of pathogenic variants in *DICER1* in young individuals with MNG. Studies have tried to estimate the prevalence of germline *DICER1* variants in women with SLCT, observing that it varies significantly from 0 to 88 % (3–9). One study has reported the frequency of germline pathogenic variants in *DICER1* in children with pleuropulmonary blastoma (PPB) to be 70 % (10). Moreover, somatic pathogenic *DICER1* variants were found in 10% (3 of 30) of papillary thyroid carcinomas belonging to individuals <18 years of age at time of diagnosis (11).

As MNG is one of the most common diseases of the thyroid gland and the most common manifestation associated with the DICER1 syndrome (12, 13), we have studied the prevalence of pathogenic germline *DICER1* variants in a Danish cohort of young individuals operated for MNG.

## Materials and Methods

### Study Enrolment

Data on individuals with SNOMED diagnosis codes nodular goitre (M71602), colloid nodular goitre (M71622) or hyperplastic nodular goitre (M71642) were obtained from the National Pathology Data Bank. The data obtained were restricted to individuals acquiring the diagnosis codes from 2001-2016 nationally in Denmark. Only individuals with a histological diagnosis and prior partial or total thyroidectomy were included. No malignant histology was found. All the included patients were ≤ 25 years at time of thyroidectomy.

**Figure 1:**
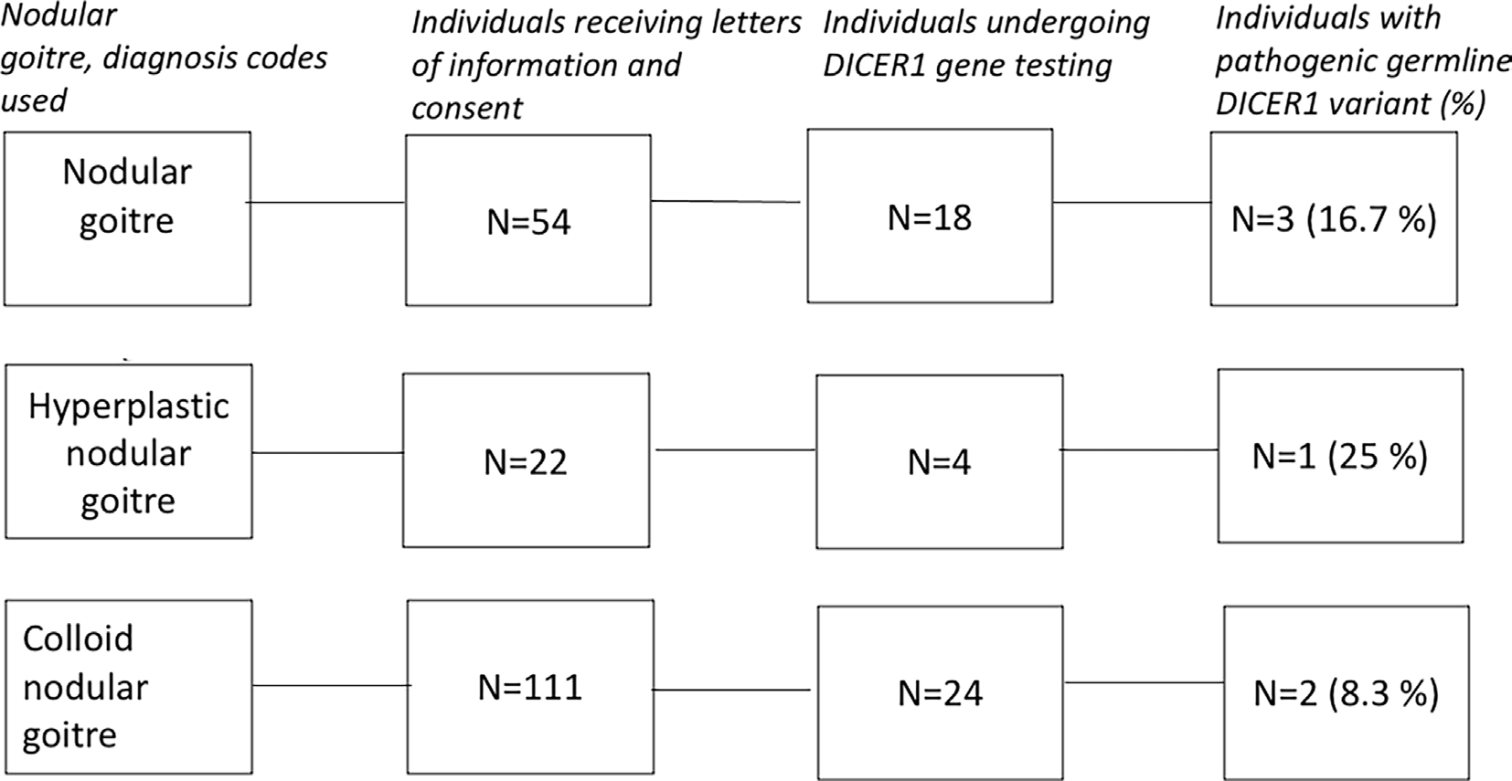
Schematic representation of the method used to obtain results.

During a period of two years (2018 and 2019), letters of information and consent were sent to all meeting the inclusion criteria except individuals who had not reached the age of 18 years; individuals who had immigrated from Denmark; and individuals who registered non-disclosure of name and address (**Figure 1**). Letters were sent to 187 individuals, albeit only eighty individuals (43 %) responded to the letter. They received oral information about the study, and information about their medical records and family history were collected. Three individuals were excluded due to a pre-existing medical condition; one excluded patient had Graves’ disease, one had a pathogenic variant in the *RET* gene and one had an unspecified non-goitre thyroid disease. Subsequently, only 46 individuals (25 %) had blood sampling performed as agreed upon.

### *DICER1* Gene Testing

Genomic DNA material was extracted from peripheral blood using standard protocols. Analysis of the coding regions and splice junctions (+/- 50 bp) of *DICER1* was performed using Next Generation Sequencing (NGS) with Illumina platforms and CNV/MLPA analysis. *DICER1* variants are numbered according to accession number NM_177438.2 using the nomenclature guidelines from the Human Genome Variation Society (https://varnomen.hgvs.org/).

The study was conducted according to the declaration of Helsinki and was approved by the Danish Data Protection Agency and Danish Regional Ethical Committee (H-17023400). Written informed consents were obtained from all participants.

## Results

Six of 46 individuals, 13%, (CI [3.3;22.7], *P* <0.05) diagnosed with nodular goitre under the age of 25 years had pathogenic germline variants in *DICER1.* All of the 40 individuals without pathogenic variants of *DICER1* had wild type alleles of the gene. Baseline characteristics of the overall nodular goitre cohort undertaking *DICER1* gene testing is shown in **Figure 1** and **Table 1**. The majority of participants undergoing *DICER1* gene testing had a family history of goitre or thyroidectomies. In the nodular goitre cohort, three individuals had a pathogenic *DICER1* variant (17%); in the colloid nodular goitre cohort, two individuals had pathogenic *DICER1* variant (8%); and in the hyperplastic nodular goitre cohort, one individual had a pathogenic *DICER1* variant (25%). The mean age at time of operation amongst individuals without pathogenic variants in *DICER1* was 19.4 years, whilst the mean age amongst individuals with pathogenic variants in *DICER1* was 15.3 years: Four of the six individuals found in the goitre cohorts carrying pathogenic *DICER1* variants had their thyroid gland totally or partially removed at < 20 years of age, the remaining two were both 21 years of age at the time of thyroidectomy (**Table 2**). Furthermore, one third of the MNG individuals with variations in *DICER1* had partial thyroidectomies – the rest had undergone total thyroidectomies. The indication for partial or total thyroidectomies was compressive symptoms due to the goitre.

**Table 1:** Baseline characteristics of the goitre cohort.

	Nodular goitre	Hyperplastic nodular goitre	Colloid nodular goitre
Number of cases (N=46)	18	4	24
Sex*: Female* (N=41)	15	4	22
* Male* (N=5)	3	0	2
Mean age	30	32	29
Thyroidectomy: *Total* (N=14, 30 %)	5 (28 %)	4 (100 %)	5 (21 %)
* Initial vs. complementary total thyroidectomy*	*3 vs. 2*	*3 vs. 1*	*2 vs. 3*
*Partial (*N=32, 70%)	13 (72%)	0 (0 %)	19 (79 %)
Mean age at total or partial thyroidectomy	19	21	17
Positive *DICER1* gene testing (N=6, 13 %)	3	1	2

**Table 2:** Characteristics of *DICER1* probands identified in the goitre cohort.

Proband	Sex	Age (y)	Type of operation	Age at time of operation (y)	DICER1 gene testing				
					*Chromosomal variant*	*Variant type*	*Protein*	*Predicted*	*Other*
							*Change*	*consequence*	*Manifestations (age)*
**1**	F	32	Total thyroidectomy	21	c.3452_3453del	Frameshift	p.(Val1151Glufs*12)	Pathogenic	Kidney cyst (14)
									Pulmonary cyst (?)
**2**	F	25	Total thyroidectomy	12	c.316del**	Frameshift	p.(Val106Leufs*22)	Pathogenic	None
**3**	F	30	Partial thyroidectomy	21	c.171_172insAC*	Frameshift	p.(His58Thrfs*8)	Pathogenic	None
**4**	F	28	Total thyroidectomy*	8	c.3434del**	Frameshift	p.(Asn1145IIefs*7)	Pathogenic	None
**5**	F	24	Partial thyroidectomy	14	c.988C>T	Nonsense	p.(Gln330*)	Pathogenic	Sertoli-Leydig Cell Tumor, (15)
**6**	F	23	Total thyroidectomy*	16	c.5388dup	Frameshift	p.(Glu1797Argfs*7)	Pathogenic	None

*Total complementary thyroidectomy, age at time of first operation.

**Variant not reported in gnomAD or in literature.

All six identified individuals with pathogenic *DICER1* variants in the goitre cohort were females. Only one of the identified individuals with pathogenic *DICER1* variants, proband 6, was already known with a germline *de novo* heterozygous frameshift, c.5388dup, p.(Glu1797Argfs*7) variant in *DICER1* (**Table 2**). The newfound proband 1 and proband 4 had no family history of goitre or other DICER1 syndrome-related manifestations. The rest of the probands had a family history of relevant manifestations.

Three of the six individuals were carriers of pathogenic *DICER1* variants not previously reported in gnomAD or in the literature: Proband 2 carried a frameshift variant, c.316del, p.(Val 106Leufs*22). Besides a total thyroidectomy performed at age 12, no other DICER1 syndrome-related manifestations were found at 25 years of age.

Proband 3 had a frameshift variant, c.171_172insAC, p. (His58Thrfs*8) in the *DICER1* gene – other than undergoing a partial thyroidectomy at age 21, her medical records were unremarkable at age 30.

Another not previously described frameshift variant was detected in proband 4, c.3434del, p.(Asn11451IIefs*7). The individual was, apart from MNG resulting in total thyroidectomy at age 11, not affected by other DICER1 syndrome-related manifestations at 28 years of age.

Two of the six individuals had manifestations related to the DICER1 syndrome other than MNG: Proband 1, carrying a frameshift variant, c.3452_3453del, p.(Val1151Glufs*12), had a pulmonary cyst and a kidney cyst measuring 11 cm surgically removed at 25 years of age. Proband 5 carried a nonsense variant in *DICER1*, c.988C>T, p.(Gln330*). She had the thyroid gland partially removed at age 14 and was additionally diagnosed with Sertoli-Leydig cell tumour (SLCT) at age 17 (**Table 2**). All the six carriers were referred to examinations in accordance with the European surveillance protocol (16) - no new manifestations related to the DICER1 syndrome have been reported.

## Discussion

To our knowledge, this is the first study estimating the prevalence of germline *DICER1* pathogenic variants among young individuals operated for MNG in an age below 25 years, finding a prevalence of 13%. Although numbers are small and the data needs to be replicated in an independent dataset, the prevalence is surprisingly high. The estimated frequency of loss-of-function *DICER1* variants in the general population is suggested to be ~ 1:10,600, which is a substantial higher number compared to the number of identified families with *DICER1* syndrome (17). This is in line with data from our study.

Considering the prevalence being 13%, we recommend referring young individuals diagnosed with goitre in need of surgery aged <21 years to genetic counselling for *DICER1* gene testing as the mean age at time of thyroidectomy amongst *DICER1* patients in this study was 15.3 years, in which the oldest ones were 21 years. Similarly, we recommend genetic counselling to individuals diagnosed with MNG in need of surgery at age <25 years, if they have at least a family history of goitre. Furthermore, referral of individuals diagnosed with MNG in need of surgery, who are affected by another DICER1 manifestation should include all age groups. Parts of the recommendations are additionally supported by one study concluding that early onset MNG or multiple cases of MNG in a family should raise concerns of *DICER1* alterations (18). Likewise, the recommendations might also apply to patients with MNG diagnosed with the support of fine needle aspiration or based on imaging (thyroid ultrasound or thyroid scintigraphy). However, as this study has explored MNG diagnosis based on histology, recommendations on *DICER1* gene testing in patients with cytology-aided or imaging-based diagnosis of MNG without the need of surgery should rely on further studies.

The six carriers of *DICER1* variants in the goitre cohort were discovered in different nodular goitre groups; nodular goitre, colloid nodular goitre, and hyperplastic nodular goitre. Different pathologists studied the different samples, which questions the consistency of the terminology use. However, this is in conjunction with the previously observed difficulty in histologically differentiation between papillary thyroid cancer and follicular thyroid cancer in DICER1 patients (15). Studies need to be conducted regarding the morphological features of nodular goitre in individuals harbouring *DICER1* variants, and whether they - in case distinguishing hallmarks can be identified - may be suggestive of DICER1 syndrome.

Somatic mutational analyses of the nodular goitre tissue samples belonging to the six patients with *DICER1* alterations were not performed. Khan et al. have reported that 84 % of thyroid nodules from 13 patients carrying germline *DICER1* variations with MNG had somatic mutations in DICER1 hotspot amino acids (2). It could have been of great value if such analyses had been executed in this study, strengthening the published data regarding the unique two-hit-mechanism and the DICER1 syndrome.

The research recruitment rate has not been high as letters of information have been sent to 187 individuals, though only 80 individuals accepted to participate and in only 46 individuals *DICER1* gene testing was performed. Almost all the participants who underwent genetic testing had a family history of goitre: This may have biased the study to a higher prevalence, assuming individuals without a family history were not interested in a study of genetics. Furthermore, most of the individuals contacted in this study were under the age of 30 years, which might also have contributed to the low participation rate, as the relevance of inherited diseases increases after having children.

All the identified DICER1 patients were females, which is to be expected as 89 % of the included individuals in the study were females. Overall, it is a well-known fact that goitre is more predominant in women (19). This is also consistent with a study based on an American cohort estimating that the incidence of thyroidectomy due to MNG is higher in women with *DICER1* variations compared to men with *DICER1* variations (2).

Total thyroidectomies were performed in four of six DICER1 patients found in the goitre cohort. Two of the four patients had initially undergone partial thyroidectomy, but, residual thyroid tissue was subsequently removed, as MNG had developed. This is in accordance with previous studies reporting that DICER1 patients undergoing partial thyroidectomy often need additional thyroid surgery (2). Furthermore, a study by Chernock et al. (20) demonstrated that poorly differentiated thyroid carcinomas in children and adolescents are strongly associated with *DICER1* mutations and are highly aggressive, although in most cases differentiated thyroid carcinomas in DICER1 patients behave in an indolent manner (20). A genome-first approach study of the *DICER1* observed that individuals harbouring putative LOF variants of *DICER1* had a significantly stronger association with thyroid cancer and thyroidectomy compared to matched controls without *DICER1* variations (21). These findings suggest considerations of early total thyroidectomy in a DICER1 patient - when goitre is detected - is relevant. However, it is also important to note that the risk of developing thyroid carcinomas secondarily to thyroid nodules has been suggested to be small (2, 22), and the cumulative incidence of thyroid cancer in carriers of *DICER1* variants remains unknown (16, 20). Studies need to be done on whether to recommend total thyroidectomy in carriers of pathogenic germline *DICER1* variants, when the diagnosis of a goitre is made, especially as the possible complications of the procedure can be postoperative hypocalcemia and recurrent laryngeal nerve paralysis (23).

**Figure 2:**
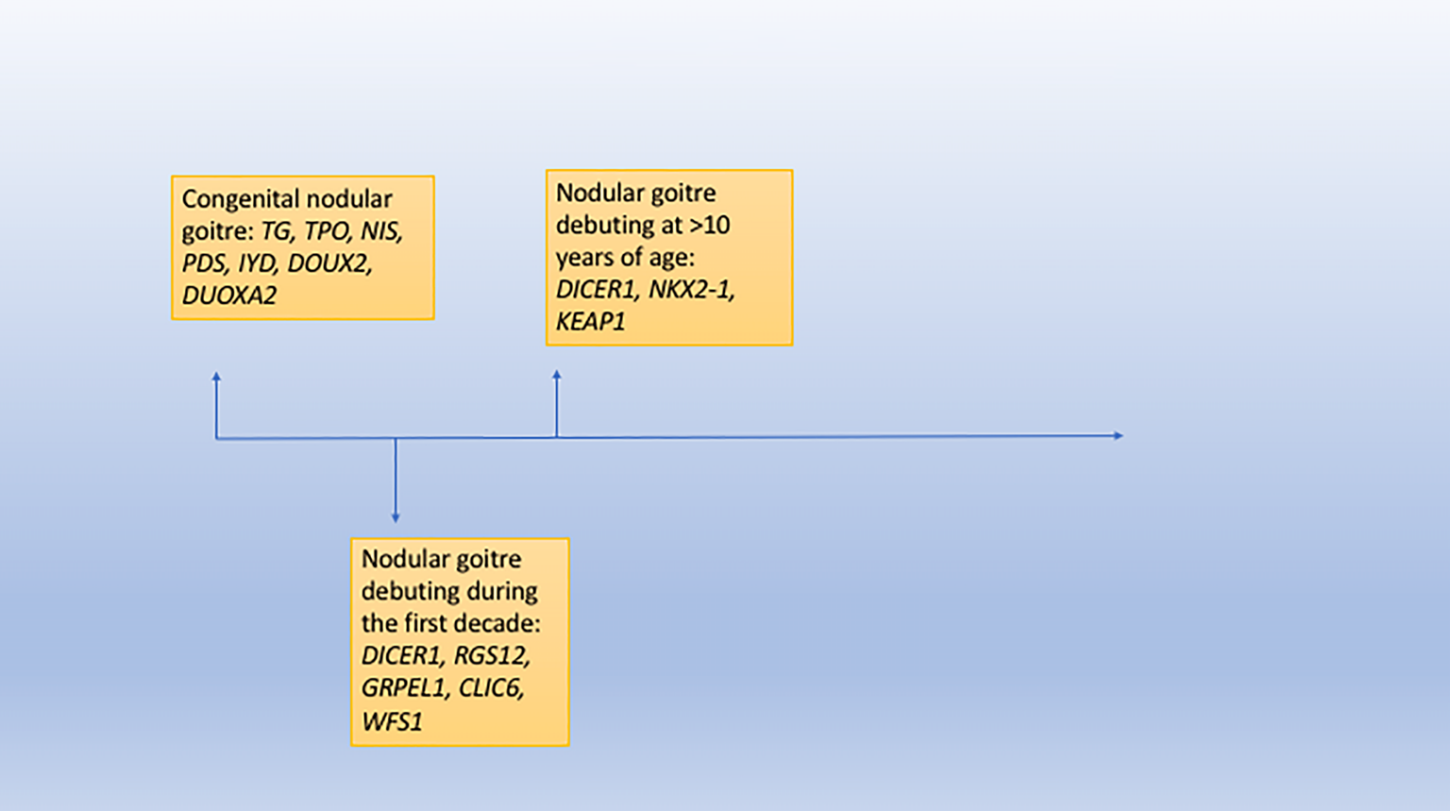
A flowchart showing age at nodular goitre diagnosis and the putative causative genes.

Familial clustering of goitre is not uncommon in areas known to be without iodine deficiency, predominately showing an autosomal dominant inheritance pattern. Variations in the genes *TG, TPO, NIS, PDS, PAX8, NKX2-1, NKX2-5, IYD, FOXE1, JAG1, DOUX2, DOUXA2* and *TSH-R* are associated with congenital hypothyroidism and/or nodular goitre (14, 24, 25). Pathogenic variants of the genes *RGS12, GRPEL1, CLIC6* and *WFS1* are suggested to be associated with an increased risk of nodular goitre, typically developing during the first decade of life – no other manifestations have been associated with the genes expect *WFS1* (Wolfram syndrome), though the literature is very sparse (26, 27). A few cases of nodular goitre due to *DICER1* alterations have been reported in the first decade of life in addition to proband 4 in this study (28), though the vast majority of cases are diagnosed in the second and third decades. Case reports show germline pathogenic variations in *KEAP1* and *NKX2-1* are also linked to nodular goitre diagnosed during the second decade of life (29, 30). **Figure 2** shows a flowchart of age at nodular goitre diagnosis along with the putative causative genes. As this study has only addressed *DICER1*, we cannot recommend testing the other candidate genes in young individuals presenting with nodular goitre, especially as some of genes are not well-studied. **Figure 2** only gives an overview of the genes presented in the literature connected to familial nodular goitre.

In conclusion, considering the estimated prevalence being 13%, we recommend referral of individuals with MNG in need of surgery aged <21 years to genetic counselling for *DICER1* gene testing. Likewise, we recommend referral of individuals with MNG in need of surgery aged <25 years with a family history of goitre to genetic counselling for *DICER1* gene testing. Individuals of all ages thyroidectomised due to goitre, who are also affected by another DICER1 syndrome-related manifestation should be referred to genetic counselling.

## Data Availability Statement

All datasets presented in this study are included in the article.

## Ethics Statement

The study was conducted according to the declaration of Helsinki and was approved by the Danish Data Protection Agency and Danish Regional Ethical Committee (H-17023400). Written informed consents were obtained from all participants. The patients/participants provided their written informed consent to participate in this study. Written informed consent was obtained from the individual(s) for the publication of any potentially identifiable images or data included in this article.

## Author Contributions

MA contributed to designing the research study, acquiring data, analysing data, and writing the original manuscript. TH contributed to acquiring data, analysing data, and revising the manuscript. ES-R contributed to analysing data and revising the manuscript. MR contributed to acquiring data. ÅR contributed to analysing data and revising the manuscript. A-MG contributed to designing the research study, acquiring data, and revising the manuscript. KW contributed to designing the research study, acquiring data, analysing data, and writing the original manuscript. All authors contributed to the article and approved the submitted version.

## Funding

The Danish Cancer Society awarded author MA a research scholarship and author KW a research grant.

## Conflict of Interest

The authors declare that the research was conducted in the absence of any commercial or financial relationships that could be construed as a potential conflict of interest.

The reviewer KT declared a past collaboration with one of the authors KW to the handling editor.

## Publisher’s Note

All claims expressed in this article are solely those of the authors and do not necessarily represent those of their affiliated organizations, or those of the publisher, the editors and the reviewers. Any product that may be evaluated in this article, or claim that may be made by its manufacturer, is not guaranteed or endorsed by the publisher.
